# Book Reviews

**Published:** 1872-04-15

**Authors:** 


### Book Reviews.

History of Medicine from the Earliest Ages to the Commencement of the Nineteenth Century. By Robley Dunglison, M. D., LL.D., late Professor of the Institutes of Medicine and of Medical Jurisprudence in the Jefferson Medical College of Philadelphia, etc., etc. Arranged and edited by Richard J. Dunglison, M.D. Philadelphia: Lindsay & Blakiston. 1872.

We have received from the publishers, through the Western News Company of this city, a copy of this work. It is a small-sized octavo volume of 287 pages, published in excellent style, and sold by subscription only, at $2.50. From a hasty glance at its pages, we think it is perhaps the best and most authentic summary of the history of medicine prior to the commencement of the Nineteenth Century, that is accessible to the profession in this country. It is concise, comprehensive, and supplied with a copious index. It is therefore well adapted for the use of both students and practitioners.

The Urine and its Derangements, with the Application of Physiological Chemistry to the Diagnosis and Treatment of Constitutional as well as Local Diseases. Being a Course of Original Lectures delivered at University College, London. By George Harley, M.D., F.R.S., Fellow of the Royal College of Physicians, etc. etc., etc. With Illustrations. Philadelphia: Lindsay & Blakiston. 1872. pp., 334. Price $2.75.

Our copy of this work was received through Jansen, McClurg & Co., booksellers, of this city; and in its mechanical execution is certainly creditable to its well-known publishers. We had thought the field embraced in this volume already well occupied by a number of able writers, whose works are familiar to the profession; but Dr. Harley has certainly given us a very valuable additional treatise, which will be found worthy of study by every practitioner who wishes to keep fully informed in regard to urinary and renal derangements. Nothing illustrates more forcibly the truly scientific character of modern medicine than the application of organic and physiological chemistry to the diagnosis and pathology of disease, as presented in works like the one before us. The author's style is concise, easy, and pleasing; and we commend the book as worth buying and reading.

When and How; or, a Collection of the more recent Facts and Ideas upon raising Healthy Children. By Dan Newcomb, M.D. Chicago: Arthur W. Penny & Co. 1872.

This is a small-sized octavo volume of 324 pages, published in fair style, and written more for the general public than the profession. It embraces eight chapters on the following topics: Physiological and Hygienic Knowledge; Like begets Like; Pure Air and Respiration; Digestion and Nutrition; Food and its Elements; Clothing and Cleanliness; Activity and Exercise; Sleep. These very important topics are discussed in a plain, practical manner; and yet so fully as to present almost every item of knowledge needed by parents in the management of their children. The author's directions in regard to all the topics named are judicious, and his style well calculated to interest the non-professional reader.

It would do much good if it could find a familiar place in every family library.

Quarterly Summary of the Transactions of the College of Physicians of Philadelphia. From May 18th, 1870, to Feb. 7th, 1872, inclusive. Philadelphia: Collins, printer, 705 Jayne street. 1872.

This is a pamphlet of 54 pages, containing an abstract of the doings of the Society for one year and eight months. The reader will find in it much of scientific and practical value.

